# The Importance of Conserved Serine for C-Terminally Encoded Peptides Function Exertion in Apple

**DOI:** 10.3390/ijms20030775

**Published:** 2019-02-12

**Authors:** Zipeng Yu, Yang Xu, Lin Liu, Yarong Guo, Xisen Yuan, Xinyu Man, Chang Liu, Guodong Yang, Jinguang Huang, Kang Yan, Chengchao Zheng, Changai Wu, Shizhong Zhang

**Affiliations:** 1 State Key Laboratory of Crop Biology, Shandong Agricultural University, Tai’an, Shandong 271018, China; yzp52120090916@163.com (Z.Y.); xy52120092661@163.com (Y.X.); 17863852581@163.com (L.L.); 15688820751@163.com (Y.G.); xisenyuan@163.com (X.Y.); shanying420@163.com (X.M.); gdyang@sdau.edu.cn (G.Y.); jghuang@sdau.edu.cn (J.H.); kangyan@sdau.edu.cn (K.Y.); cczheng@sdau.edu.cn (C.Z.); 2 Shandong Peanut Research Institute, Shandong Academy of Agricultural Sciences, Qingdao 266100, China; 3 Entomology and Nematology Department, University of Florida, Gainesville, FL 32611, USA; c.liu@ufl.edu

**Keywords:** bioinformatics, C-terminally encoded peptide, *Malus domestica*, phylogenetic analysis, root growth, secreted peptides

## Abstract

Background: The C-terminally encoded peptide (CEP) family has been shown to play vital roles in plant growth. Although a genome-wide analysis of this family has been performed in Arabidopsis, little is known regarding CEPs in apple (*Malus domestica*). Methods: Here, a comprehensive bioinformatics approach was applied to identify MdCEPs in apple, and 12 *MdCEP* genes were identified and distributed on 6 chromosomes. Results: MdCEP1 peptide had an inhibitory effect on root growth of apple seedlings, indicating that MdCEP1 played a negative role in root development. In addition, the serine and glycine residues remained conserved within the CEP domains, and MdCEP1 lost its function after mutation of these two key amino acids, suggesting that Ser^10^ and Gly^14^ residues are crucial for MdCEPs-mediated root growth of apple. Encouragingly, multiple sequence alignment of 273 CEP domains showed that Ser^10^ residue was evolutionarily conserved in monocot and eudicot plants. MdCEP derivative (Ser to Cys) lost the ability to inhibit the root growth of *Nicotiana benthamiana*, *Setaria italic*, *Samolous parviflorus*, and *Raphanus sativus* L. and up-regulate the NO_3^−^_ importer gene *NRT2.1*. Conclusion: Taken together, Ser^10^ residue is crucial for CEP function exertion in higher land plants, at least in apple.

## 1. Introduction

Cells within higher plants must participate in both short and long ranges cell–cell communication to ensure proper patterning and functional connections [1,2,3]. The process is achieved through a series of substances, mainly including phytohormones and small secreted peptides [4,5]. Most studies have focused primarily on phytohormones, while during the last decade, the importance of secreted peptides in plant cell-to-cell communication, growth and development has become increasingly clear [6,7,8,9]. The secreted peptides fall into one of two broad groups: the cysteine-rich peptides (CRPs) and post-translationally modified peptides (PTMs) [4]. CRPs as a highly abundant, divergent and important class of functional peptides are characterized by the presence of a C-terminal cysteine-rich domain [10]. The even number of cysteine residues in CRPs are required for the formation of disulfide bonds that maintain the mature peptide in a bioactive conformation [10,11,12]. The other group, PTMs, often undergo proteolytic processing, and are processed into bioactive mature peptide with approximately 20 amino acids (aa) in length [4,13].

An in silico approach, specifically Perl scripts, was performed to identify genes encoding peptides with N-signal peptide. This led to the discovery of the C-terminally encoded peptide (CEP) family in Arabidopsis [14]. The mature products underwent N-signal peptide proteolytic cleavage from the expressed precursor as PTMs and were processed into a 15 aa peptide containing one or two hydroxylated proline residues [14].

Over the past decade, various studies have emerged implicating CEPs to be involved in a wide range of biological processes, including systemic N-demand signaling and plant growth and development [14,15,16]. CEPs originating from the N-starved roots are translocated from root to shoot and concentrated within the leaf vascular bundles, which are recognized by two leucine-rich repeat receptor-like kinases, CEP receptor 1 (CEPR1) and CEPR2 [16,17]. Then the shoot-derived polypeptides, CEP DOWNSTREAM 1 (CEPD1) and CEPD2, act as descending long-distance mobile signals downstream of the CEP-CEPR ligand-receptor system to translocate from shoot to root and up-regulate the expression of the nitrate transporter gene *NRT2.1*, and eventually uptake nitrate in root and mediate systemic N-demand signaling in plants [18]. Moreover, CEP peptides also play a vital function in plant growth and development. AtCEP1 arrests primary root and lateral root growth through repression of meristematic cell division and expansion, and AtCEP5 assists in the lateral root and aboveground development [14,19]. Moreover, AtCEP3 mediates root and shoot development in response to environmental cues [20].

Apple tree is one of the most important and economical woody plants widely cultivated throughout the temperate zones [21]. The main function of the CEP family in Arabidopsis is to promote the absorption of environmental nitrogen sources by roots, the crucial issue of apple. In addition, small signaling peptides play a crucial role in many stages of plant development. The identification and study of MdCEPs provides important clues to understand the molecular mechanism of apple root growth and its response to diverse environmental factors. The new whole genome of apple has been sequenced in 2017 (GDDH13 version 1.1 database, https://iris.angers.inra.fr/gddh13/the-apple-genome-downloads.html) (access on 15 January 2018), offering an excellent opportunity for the comprehensive analysis of *MdCEPs* across the genome [22]. This study identified the MdCEP family in apple, and analyzed the contribution of the conserved amino acid to the activity of MdCEP.

## 2. Results

### 2.1. Identification and Phylogenetic Analysis of MdCEPs in Apple

To identify MdCEPs in apple, an in silico search was performed on the apple genome database GDR (Genome Database for Rosaceae: http://www.rosaceae.org/) (access on 15 January 2018) using the protein sequences of the Arabidopsis CEP domains hidden Markov model (HMM) profile (PF00319) as a query for BLAST searches. 12 MdCEPs were identified contained an N-terminal signal peptide, a C-terminal CEP-like domain, and a total length of 75–250 aa in the apple genome (Supplementary Figure S1). In an attempt to determine the reliability of the screening members, the protein sequences of the putative MdCEPs were searched for the presence of the CEP domain using Pfam and NCBI-CDD databases again, and all 12 members belonged to the MdCEP family.

**Figure 1:**
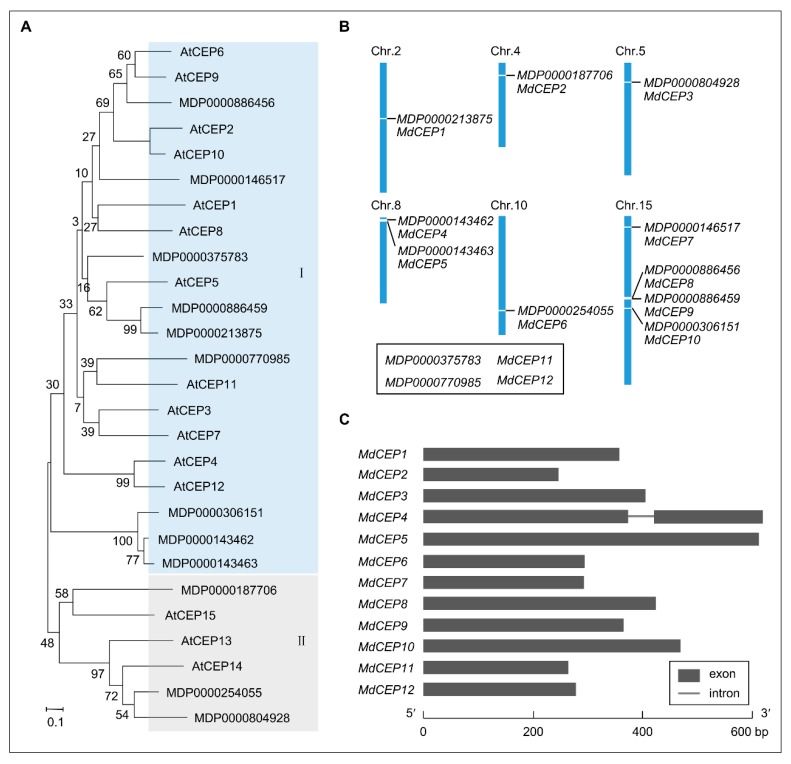
Identification and phylogenetic analysis of MdCEPs in apple. (**A**) The phylogenetic relationship of 15 AtCEPs and 12 MdCEPs. The phylogenetic tree based on CEP domain is created with MEGA7 by the neighbor-joining method and the bootstrap tests are indicated on the tree. CEP, C-terminally encoded peptide. (**B**) The chromosome location of *MdCEPs* in apple. The unanchored contigs are marked in the open squares. *MdCEP11* and *MdCEP12* are situated on unanchored contigs (Apple’s whole genome sequencing is incomplete). (**C**) The gene structures of *MdCEPs* in apple.

To further gain insights into evolutionary relationships among CEPs and to group them within the established subfamilies, protein sequences of MdCEPs and AtCEPs were subjected to multiple sequence alignment with the MEGA7 program. The multiple sequence alignment file was subsequently used to construct an unrooted phylogenetic tree using the neighbor-joining (NJ) method (Figure 1A). Furthermore, MdCEPs could be divided into two groups, where 9 members (MdCEP1–MdCEP9) were unambiguously classified as type Ⅰ, and the remaining 3 members (MdCEP10–MdCEP12) were type Ⅱ in apple.

Analysis of the chromosomal location showed that 12 *MdCEP*s were mapped on 6 chromosomes (Chr. 2, 4, 5, 8, 10 and 15) at different densities (Figure 1B). However *MdCEP11* and *MdCEP12* were situated on unanchored contigs (Apple’s whole genome sequencing is incomplete) (Figure 1B). Particular information concerning *MdCEPs* was identified and listed in Supplementary Table S1. The corresponding coding sequences of *MdCEPs* range from 246 to 615 base pairs with deduced protein sizes ranging from 81 to 204 aa. The molecular weight and isoelectric point of MdCEPs ranged from 9183.6 Da (MdCEP2) to 20,643.54 Da (MdCEP5) and from 5.87 (MdCEP7) to 10.62 (MdCEP12), respectively. Structural analysis of the *MdCEPs* showed that except for *MdCEP4*, which has two exons and one intron, the other *MdCEPs* universally lacked introns, and displayed simple structures with one exon (Figure 1C).

### 2.2. Signal Peptide Cleavage Sites Prediction and Promoter Analysis of MdCEP Members

**Figure 2:**
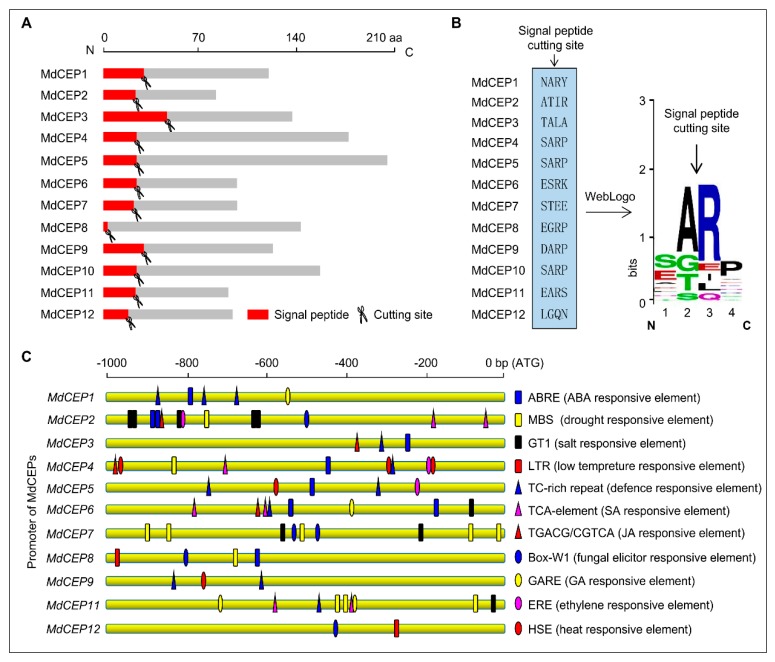
Signal peptide cleavage sites prediction and promoter analysis of MdCEP members in apple. (**A**) Signal peptide of MdCEPs deduced with SignalP 4.1 website. The red boxes represent signal peptides and the scissors are predicted signal peptide cleavage sites. (**B**) The predicted signal peptide cleavage site. A WebLogo representation of the cleavage site is shown in the right image. (**C**) Analysis of various stress response cis elements in the promoter regions upstream of *MdCEPs* via the PlantCARE website. Eleven cis elements are displayed in different patterns.

Small secreted peptides often undergo N-terminus signal peptide proteolytic processing to generate the mature peptides [23]. As the typical secreted peptide, the presence and location of the signal peptide cleavage sites in MdCEP pre-propeptides were predicted using SignalP 4.1 software (Supplementary Figure S2 and Figure 2A), and it is likely that the cleavage occurs at a conserved arginine (Figure 2B).

Since the temporal, spatial, and cell type-specific control of gene expression mainly depend on cis regulatory elements, the promoter regions of *MdCEPs* (except *MdCEP10*, Apple’s whole genome sequencing is incomplete) were isolated and analyzed in the PlantCARE website. As shown in Figure 2C, a number of cis-acting elements related to stresses as well as phytohormone responses were found in the promoter regions. Defense-related elements such as TGACG/CGTCA (jasmonic acid response element), TCA element (salicylic acid response element), ERE-element (ethylene response element), TC-rich repeat (defense responsive element), Wun motif (wound response element), and BOX-W1 (fungal elicitor-responsive element) were discovered in promoter regions of more than three *MdCEPs*. Abiotic stress response element, MYB-binding sites (MBSs) and ABA-responsive elements (ABREs) were abundant in *MdCEP* promoters. Moreover, *MdCEP* members also contained other stress response elements: GT1 (GAAAA, salt stress response element), HSE (heat stress response element), LTR (low-temperature response element) and GARE (GA response element). These results indicate that *MdCEPs* may play potential roles in stress response in apple.

### 2.3. MdCEPs Display Distinct Spatial Expression Patterns in Apple

**Figure 3:**
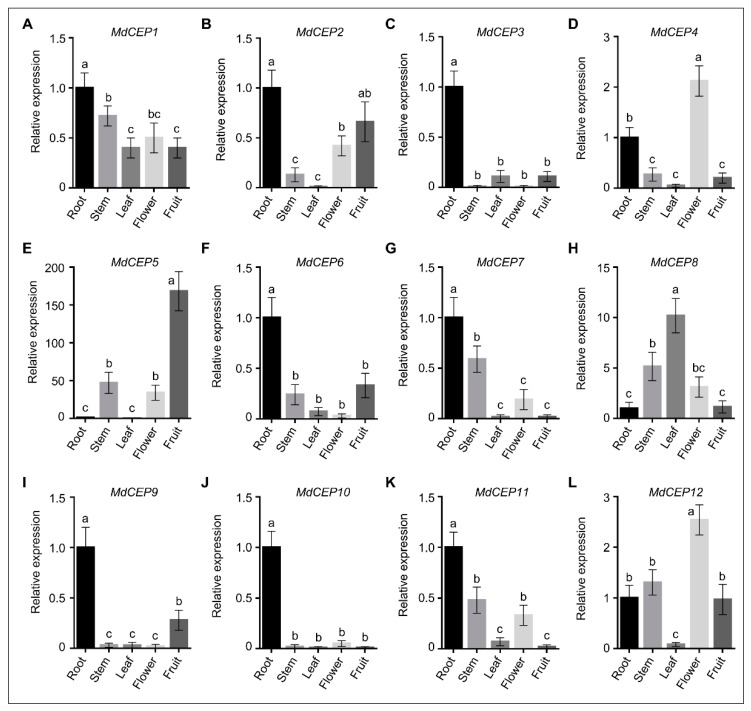
The tissue expression pattern of MdCEPs in apple. The expression levels of *MdCEP1*–*MdCEP12* (**A**–**L**) examined by qRT-PCR in various tissue and stages in apple. The *18s rRNA* was performed as an internal control. Relative abundance of *MdCEP1*–*MdCEP12* transcripts from various tissue and stages represented normalized against *18s rRNA*. Error bars indicate SEM (*n* = 3), *p* < 0.05. One-way ANOVA Duncan’s test is used for statistical analysis. These experiments were repeated three times with similar results. *rRNA*, *ribosomal RNA*.

The gene spatial expression pattern can provide important clues for investigating gene function. In this study, quantitative RT-PCR (qRT-PCR) was performed by using roots, stems, siliques, flowers and leaf tissues of *Malus hupehesis* (Pamp.) Rehd to gain further insight in the expression patterns of *MdCEPs* in apple. 12 *MdCEPs* could be largely divided into two groups: root higher expression members and shoot higher expression members. Expressions of eight *MdCEPs*, including *MdCEP1*, *2*, *3*, *6*, *7*, *9*, *10* and *11*, occur preferentially in roots, which might be associated with root growth function in apple (Figure 3). *MdCEP4* and *MdCEP12* showed higher expression levels in flowers compare to roots, making these likely candidates for controlling flower development (Figure 3D,L). For the other two *MdCEP* genes, high mRNA level of *MdCEP5* was observed in fruits, and *MdCEP8* was mainly expressed in leaves and stems (Figure 3E,H). The diverse expression levels in various tissues imply that *MdCEPs* may play distinct physiological and developmental roles during various developmental processes.

### 2.4. MdCEPs Involved in Multiple Biotic and Abiotic Stresses Response

**Figure 4:**
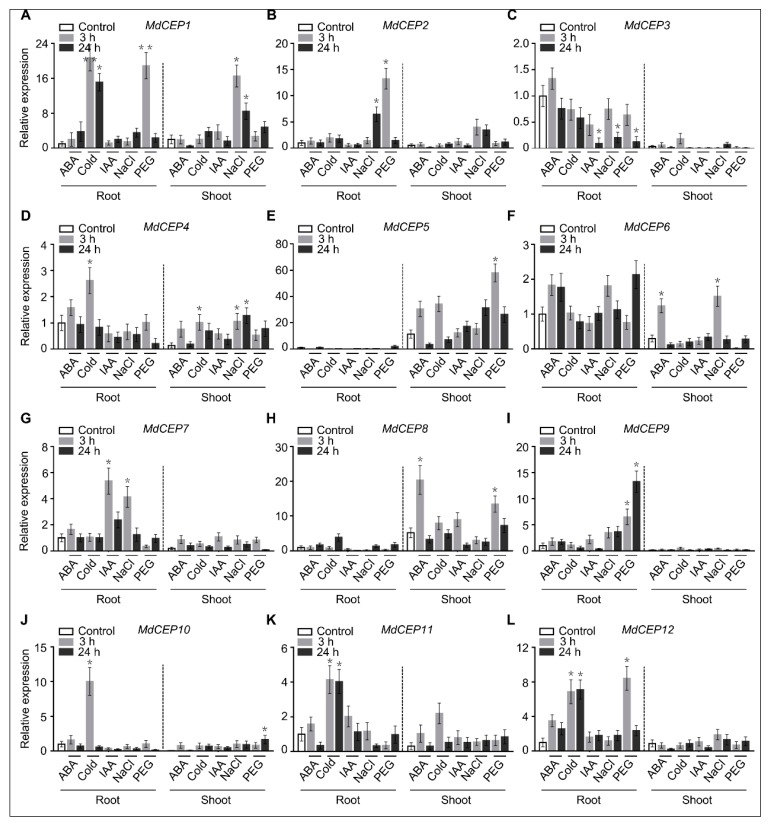
The expression pattern of *MdCEPs* under stress treatments in apple. Fourteen-day-old seedlings of *Malus hupehesis* (Pamp.) *Rehd* supplied with different treatments and the aboveground parts and roots are collected for RNA extraction and qRT-PCR analysis. (**A**–**L**) Representation of the transcription levels of *MdCEP1*–*MdCEP12*. Relative abundance of *MdCEP1*–*MdCEP12* transcripts from various treatments represented as fold change relative to mock-treated values and normalized against *18s rRNA*. Error bars indicate SEM (*n* = 3). * *p* < 0.05; ** *p* < 0.01 (Student’s *t*-test). These experiments were repeated three times with similar results.

To elucidate the potential roles of *MdCEPs* in plant stress response, wild-type *Malus hupehesis* (Pamp.) Rehd with several treatments were collected and performed with induction experiments. Expression of *MdCEP11* was especially increased in cold-treated plants, indicating that *MdCEP11* could be involved in cold stress response (Figure 4K). Transcript levels of *MdCEP1* and *MdCEP12* were enhanced by cold and PEG treatment in root and salinity treatment in shoot (Figure 4A,L). Expression of *MdCEP5* was apparently increased by PEG treatment in shoot, and *MdCEP6* and *MdCEP8* were also exclusively induced by ABA treatments in shoot, which was in accordance with the aboveground apart specific expression pattern (Figure 4E,F,H). In contrast, a root-special expressed member, *MdCEP7* was upregulated significantly in root by IAA and high salinity treatments (Figure 4G). *MdCEP2* and *MdCEP9* had similar expression pattern and were transcriptionally up-regulated by salinity and PEG treatments, whereas *MdCEP3* expression was down-regulated dramatically by salinity and PEG treatments (Figure 4B,C,I). In addition, cold treatment increased the expression of *MdCEP4* and *MdCEP10* in root, whilst they could be induced by salinity and PEG in shoot, respectively (Figure 4D,J). Thus we speculate that the functional variance of *MdCEPs* may play distinct roles in various stresses response in apple.

### 2.5. Distinct Post-Translational Modifications of MdCEPs in Apple

**Figure 5:**
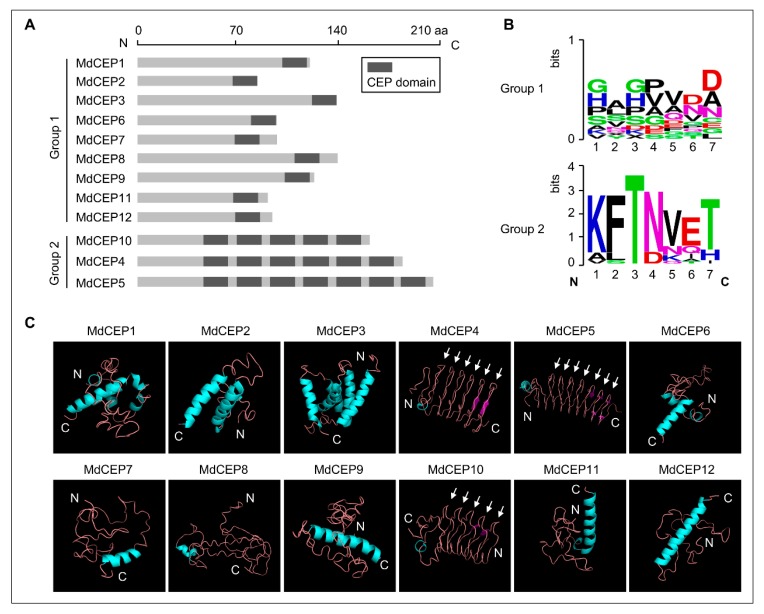
MdCEPs are divided into 2 subgroups according to the number of CEP domains. (**A**) Schematic representation of MdCEP family members. Group 1 contains members with one CEP domain; Group 2 contains five-seven CEP domains. The dark grey boxes represent CEP domains. (**B**) A WebLogo representation of the sequence before CEP domains (Group 1) and adjoining sequence between CEP domains (Group 2). (**C**) Three-dimensional structures of MdCEPs predicted by I-TASSER website. The CEP domains in Group 2 are pointed by arrows.

Protein multiple sequence alignments for Arabidopsis and apple revealed that they encode an N-terminal secretion signal and consist of CEP domains (Figure 2A and Figure 5A). Similar to AtCEPs, the CEP domain occurs multiple times within one pre-propeptide in several MdCEPs (Figure 5A and Supplementary Figure S1) [15]. According to the number of CEP domains, MdCEPs could also be divided into two groups, group 1 consisted of 9 MdCEP members (MdCEP1, 2, 3, 6, 7, 8, 9, 11, 12), which has one CEP domain at the C-terminus, and the remaining members (MdCEP4, 5 and 10) are classified as group 2, which contains five to seven CEP domains (Figure 5A).

CEPs often undergo post-translational modifications and processed into biologically active peptides via proteolytic processing [23]. Sequences on both sides of the CEP domain might contain potential recognition sites of the proteolytic enzymes. Multiple sequence alignments showed that the MdCEP members in group 1 had little sequence conservation, while the adjoining sequences (KFTNVET) between CEP domains in group 2 showed high amino acid similarity (Figure 5B and Supplementary Figure S5). Thus, some special proteolytic enzymes might recognize the certain amino acid in the links (KFTNVET) between CEP domains of group 2 members, whereas the proteolytic enzymes recognized the group 1 members might be variant. Furthermore, three-dimension structure analysis revealed that MdCEP members in group 2 shared similar spatial structure with several regular coils associated with the number of CEP domains, and MdCEP members in group 1 contained one CEP domain (*α*-helix) in the C-terminus (Figure 5C). Apart from the CEP domains themselves, the other structures of MdCEPs indicated disorder with diverse features (Figure 5C). Thus, MdCEP peptides might undergo at least two kinds of proteolytic processing in apple.

### 2.6. The Conserved Serine and Glycine Are Critical for Function Exertion of MdCEPs

**Figure 6:**
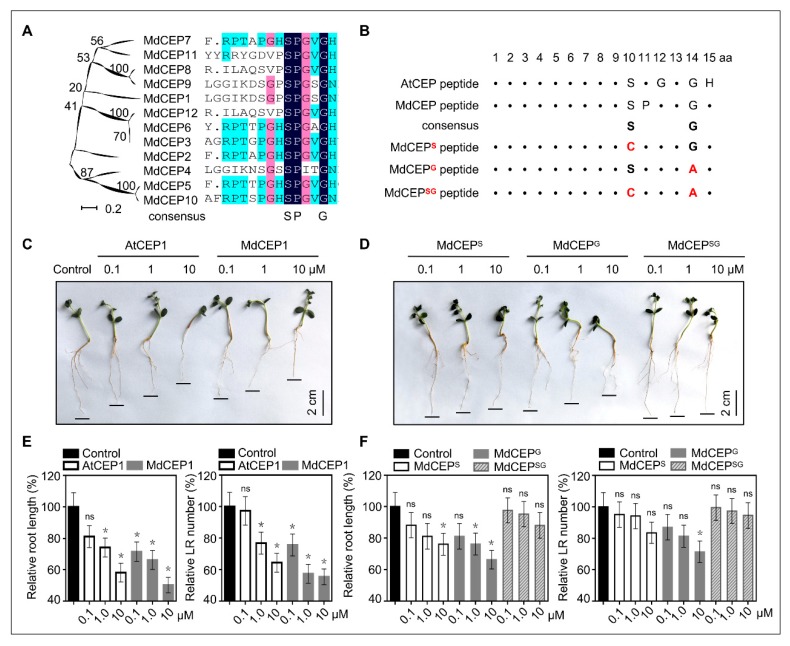
The conserved serine and glycine are critical for MdCEPs in root growth. (**A**) Multiple alignment of the CEP domains from 12 MdCEP members. Overall conserved amino acids are in black. (**B**) Conserved amino acids analysis of all MdCEPs and AtCEPs. Different substitution derivatives are obtained by changing the conserved amino acids indicate in red. (**C**) Inhibitory effects of exogenous AtCEP1 and MdCEP1 peptide on root growth of apple seedlings. Apple seeds were plated on MS medium with or without different CEP peptides and grown for 2 weeks. (**D**) Inhibitory effects of exogenous substitution derivatives of MdCEP1 on root growth of apple seedlings. Apple seeds were plated on MS medium with or without different CEP peptides and grown for 2 weeks. (**E**,**F**) The relative primary root length and lateral root number of various plants in (**C**,**D**). Error bars indicate SEM (*N* = 3, *n* = 24). * *p* < 0.05; ns, no significance. (Student’s *t*-test).

Surprisingly, apart from the N-terminus secretion signals and the CEP domains themselves, CEPs displayed little sequence conservation (Supplementary Figure S1). This indicated that the function exertion of CEPs might depend on one or several specific amino acids in the CEP domain, especially the conservative amino acid(s). Then this work used all the MdCEP domains to perform sequence comparison and phylogenetic analysis, which revealed three conversed residues (serine, proline, and glycine) were discovered in all the 12 MdCEPs (Figure 6A). Subsequently, to analyze the conservative feature of these amino acids, amino acid sequences of 12 MdCEPs and 15 AtCEPs were further subjected to multiple sequence alignment, and two conserved residues (serine and glycine) were retained in apple and Arabidopsis (Figure 6B).

To further investigate the contribution of these two conserved amino acids to the biological activity of MdCEP, we obtained five kinds of chemically synthesized peptides, MdCEP1, MdCEP^S^ (Ser^10^ changed to Cys^10^), MdCEP^G^ (Gly^14^ changed to Ala^14^), MdCEP^SG^ (Ser^10^ and Cys^10^ were both changed) and AtCEP1 (positive control), and various apple seeds were subject to treatment with these peptides. AtCEPs have been described to play roles in root growth and development in Arabidopsis [14]. In our study, AtCEP1 and MdCEP1 could dramatically arrest the primary root growth and lateral root development of apple seedlings, indicating that the chemically synthesized AtCEP1 and MdCEP1 have biological activity and played a similar role in root development (Figure 6C,E). Interestingly, the single substitution derivatives MdCEP^G^ could still inhibit primary root growth and lateral root development, whereas the inhibitory effect of MdCEP^S^ was much smaller than MdCEP1 (Figure 6D,F). Additionally, the primary root growth and lateral root development were slightly inhibited by MdCEP^S^ (Figure 6D,F), suggesting that the conserved serine was the critical residue in the CEP domain. However, this inhibitory effect of MdCEP^SG^ was totally lost (Figure 6D,F), suggesting that the glycine also exerted a certain role in root growth. Taken together, the conserved serine and glycine residues are critical for function exertion of MdCEPs in root growth.

### 2.7. MdCEP Overexpression Leads to a Retarded Growth in Apple Callus

**Figure 7:**
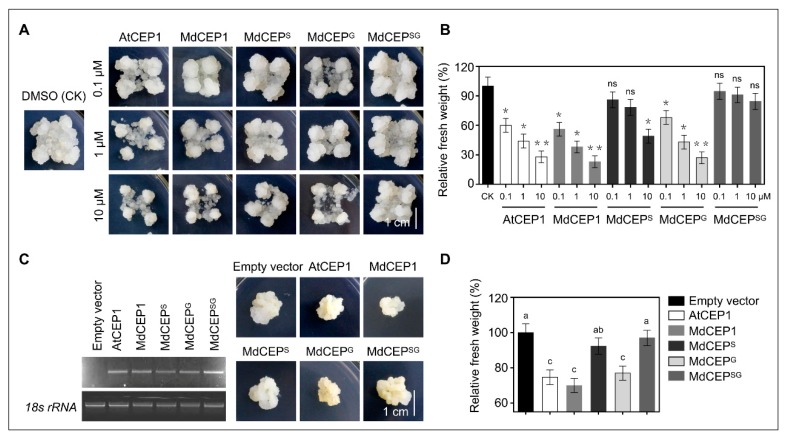
MdCEP overexpression leads to a retarded growth in apple callus. (**A**) Ten-day-old apple callus with similar size were cultured on different concentrations of CEPs (AtCEP1, MdCEP1 and substitution derivatives of MdCEP1). Subsequently, these callus were grown for another 18 d under continuous dark conditions. (**B**) The fresh weight of various callus in (**A**). Error bars indicate SEM (*N* = 3, *n* > 30). * *p* < 0.05; ** *p* < 0.01 (Student’s *t*-test). Statistical analysis is based on three independent biological repeats. ns, no significance. (**C**) The phenotype of transgenic and non-transgenic apple callus (empty vector, control) grown on MS medium for 18 d. And the expression levels of *AtCEP1*, *MdCEP1*, *MdCEP^S^*, *MdCEP^G^*, and *MdCEP^SG^* were detected by RT-PCR. (**D**) The fresh weight of transgenic and non-transgenic apple callus in (**C**). Error bars indicate SEM (*N* = 3, *n* > 45), *p* < 0.05. One-way ANOVA Duncan’s test was used for statistical analysis. Statistical differences are indicated by lowercase letters and different letters represent different significance. These experiments were repeated three times with similar results.

The 10-day-old individual genotype apple callus were cultured on different concentrations of chemically synthesis CEPs. Subsequently, these callus were grown for another 18 d under the continuous dark conditions. As shown in Figure 7A,B, the apple callus exposed to AtCEP1 and MdCEP1 exhibited obvious retarded growth, whereas no statistically significant differences were discovered in callus between MdCEP^SG^ and control.

To further gain insights of the function of MdCEPs, overexpression constructs of various CEPs were transformed into apple callus through *Agrobacterium*-mediated genetic transformation. Then, the expression levels of *AtCEP1*, *MdCEP1*, *MdCEP^S^*, *MdCEP^G^*, and *MdCEP^SG^* were detected and confirmed by qRT-PCR (Figure 7C). The AtCEP1, MdCEP1 and MdCEP^G^ overexpression callus appeared smaller than did the MdCEP^S^ and MdCEP^SG^ callus (Figure 6C,D). Therefore, MdCEPs are involved in plant growth and development in apple, and serine was the master functional amino acid of MdCEPs.

### 2.8. Serine is Evolutionarily Conserved in Higher Plants and MdCEP^S^ Loses the Role in Inhibiting Root Growth

**Figure 8:**
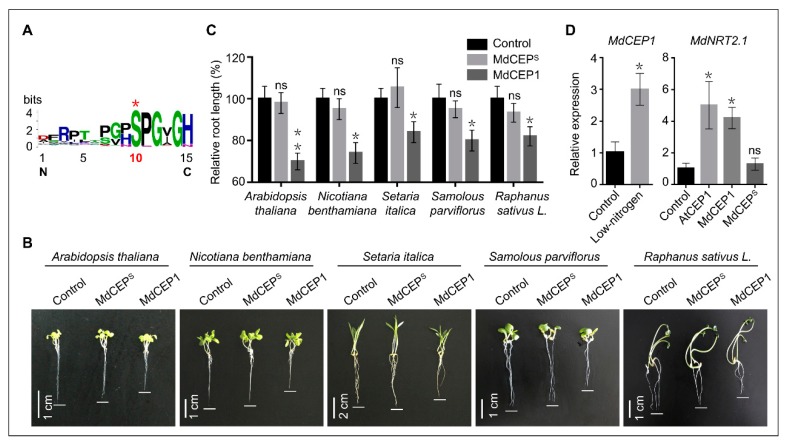
The serine is evolutionarily conserved in 32 higher land plants. (**A**) The evolutionarily conserved amino acid of CEPs in higher land plants. A WebLogo representation of the sequence of 273 CEP domains in 32 plant species. (**B**) Inhibitory effects of exogenous MdCEP1 and MdCEP^S^ on growth of *Arabidopsis thaliana*, *Nicotiana benthamiana*, *Raphanus sativus* L., Gramineae (*Setaria italica*) and *Setaria italica* (*Brassica campestris* L.). These sterile seeds were plated on MS medium with MdCEP1 or MdCEP^S^ and grown for 2 weeks. *Arabidopsis thaliana* was used as the positive control to detect the activity of the synthesized MdCEP1. (**C**) Relative primary root length of the higher land plants shown in (**B**). Error bars indicate SEM (*N* = 3, *n* =30). * *p* < 0.05; ** *p* < 0.01 (Student’s *t*-test). ns, no significance. (**D**) The relative expression levels of *MdCEP1* in 14-day-old apple seedlings treated with 0.5 mM nitrates. And the relative expression levels of *MdNRT2.1* in 14-day-old apple seedlings treated with AtCEP1, MdCEP1 or MdCEP^S^. Error bars indicate SEM (*N* = 3). * *p* < 0.05 (Student’s *t*-test). ns, no significance.

In view of the critical function of serine in MdCEP in apple, we aimed to further characterize the function of serine in CEP domain of the super-group Plantae. Comparative analysis revealed orthologues of CEPs could be found only in monocot and eudicots, but were absent in single-celled algae or other lower land plants (Fern and Bryophyte) (Supplementary Figure S3). Multiple sequence alignment of 273 CEP domains in 32 plant species showed that Ser^10^ was evolutionarily conserved in monocot and eudicot plants (Figure 8A). The elongation of the crown roots of *Arabidopsis thaliana* (used as the positive control), *Nicotiana benthamiana*, Gramineae (*Setaria italica*), *Samolous parviflorus*, *Raphanus sativus L.* were significantly arrested by chemically synthesized MdCEP1, which were reduced by 20–30% compared with those of seedlings under normal conditions (Figure 8B,C). However, the crown roots of these seedlings treated with MdCEP^S^ were statistically indistinguishable from that of control (Figure 8B,C).

Moreover, various studies have emerged implicating CEPs to be involved in systemic N-demand signaling, which can up-regulate the expression of the nitrate importer gene *NRT2.1* and uptake nitrate in roots in the N-starved plants [18]. Firstly, the *MdCEP1* gene was up-regulated under the low-nitrogen condition, supporting the function of MdCEP1 in N-demand signaling pathway in apple (Figure 8D). Secondly, the nitrogen transporter gene *MdNRT2.1* was up-regulated in apple seedlings treated with MdCEP1 rather than MdCEP^S^, suggesting that the conserved serine was an indispensable residue for MdCEP1 in systemic N-demand signaling (Figure 8D). Altogether, MdCEP1 participates in root development and the N-demand signaling pathway, and the conserved serine is crucial for CEP function exertion in higher land plants, at least in apple.

## 3. Discussion

Various studies have emerged implicating members of the CEP family, one class of PTMs that is involved in a wide range of cellular growth and development processes [24]. A comprehensive bioinformatics approach was applied to identify the MdCEP family and to determine the mode of action of this family in apple (Figure 1, Figure 2, Figure 3, Figure 4 and Figure 5); Analysis of the function of MdCEPs revealed that serine played an indispensable role in CEP function exertion (Figure 6 and Figure 7); Finally, comparative analysis of apple CEPs with other higher land plants CEPs indicated that CEPs might have similar functions in various species (Figure 8 and Supplementary Figures S3–S5). There are four conserved amino acids in AtCEPs, while there are three in MdCEPs, and only two of them are completely consistent (G and S), which indicates that the specific functions of AtCEPs and MdCEPs may be different. Moreover, the CEP mature form in Arabidopsis has been proved to be a small peptide with 15 aa, but the CEP mature form in apple has not been identified. The mature peptide of CEPs in apple should be verified by mass spectrometry.

Secreted peptides often undergo post-translational modifications to process into the mature peptides [23,25]. These CEP peptides are initially translated as pre-propeptides, then signal peptidase can recognize the cleavage sites and remove the N-terminal signal peptide to yield propeptides. After that, hydrolytic enzymes cut the propeptides to produce mature secreted peptides [8]. As illustrated in Figure 5, MdCEPs in group 2 might be recognized via the special proteolytic enzymes due to the sequence (KFTNVET) showing high similarities. However, the proteolytic processing might be diverse between different MdCEPs in group 1 because of the disordered sequences.

Notwithstanding the fact that the CEP domain is short with 15 amino acids long, previous study has shown that the finally mature CEP domain after post-translational modifications thought to be enough to exert roles [14]. However, only one amino acid residue exhibited highly conserved patterns in 273 CEP domains in 32 higher land plants (Figure 6B and Supplementary Figure S4). The sequence alignment suggested that the conserved amino acid residue might be indispensable for CEPs, and our results further verified the assumption that serine was of the essence in the exertion of their function in plant growth regulation (Figure 6, Figure 7 and Figure 8). Serine may be essential for maintaining the correct three dimensional structure and active conformation of CEPs, which may be crucial for being recognized by specific receptors.

Multiple sequence alignment of adjoining sequences between CEP domains in 11 plant species showed that the linker (KFTNVET) showed high amino acid similarity, suggesting that the proteolytic enzymes and the mature processing of CEPs may be same or similar in these monocot and eudicot plants (Supplementary Figure S5). Moreover, the conserved 7-amino-acid linker sequence between CEP domains in higher land plants may hint to a specific mechanism for these small signaling peptides, which need to explore in the future. Previous study has shown that mature active peptide contains the canonical 15 amino acids CEP domain thought to be enough to exert roles [14], and further phylogenetic data indicated that members from apple, Arabidopsis and other higher land plants demonstrated relatively high sequence identities in CEP domains. Thus CEP family may exert highly conserved role in inhibiting primary root growth, arresting the lateral root development and promoting the absorption of NO_3^−^_ in higher land plants due to the high sequence and mature processing identities.

## 4. Materials and Methods

### 4.1. Identification of MdCEPs in Apple

To identify the members of *MdCEPs*, searches of multiple databases were performed in stepwise. AtCEPs protein sequences for Arabidopsis were used as queries to perform repetitive blast searches against the GDR database (Genome Database for Rosaceae: http://www.rosaceae.org/) (access on 15 January 2018). Furthermore, the predicted sequences of *MdCEPs* were downloaded in AppleGFDB software (Apple Gene Function and Gene Family Database: http://www.applegene.org/) (access on 15 January 2018). All protein sequences derived from the candidate *MdCEP* genes were collected and examined with the domain analysis programs Pfam (Protein family: http://pfam.sanger.ac.uk/) (access on 18 January 2018) and NCBI Conserved Domain Search (http://www.ncbi.nlm.nih.gov/Structure/cdd/wrpsb.cgi) (access on 20 January 2018) with default cutoff parameters [26,27]. Isoelectric points and molecular weights of MdCEPs were obtained via proteomics and sequence analysis tools available on the ExPASy Proteomics Server (http://expasy.org/) (12 March 2018) [28]. All software and websites used are listed in Supplementary Table S2.

### 4.2. The Chromosomal Location and Gene Structures of MdCEPs

Chromosomal location and gene structures of *MdCEPs* were downloaded from the GDR database. Chromosomes locations were drawn with MapDraw59, and the gene structures were generated with GSDS (Gene Structure Display Server: http://gsds.cbi.pku.edu.cn/) (access on 15 March 2018). All software and websites used are listed in Supplementary Table S2.

### 4.3. Sequence Alignment and Phylogenetic Analysis

MdCEP sequences were aligned with the program ClustalX using BLOSUM30 as the protein weight matrix, and MUSCLE (Multiple Sequence Comparison by Log-Expectation) program was additionally used to perform multiple sequence alignments to confirm ClustalX data output (http://www.clustal.org/) (access on 25 January 2018) [29]. Phylogenetic trees of MdCEP protein sequences were constructed with the NJ method of the program MEGA7 with p-distance and complete deletion option parameters engaged. Reliability of the derived tree was tested using bootstrapping with 1,000 replicates as the previous study [30]. To further characterize the function of CEPs in the super-group Plantae, we assessed the presence or absence of CEPs in 39 plants using the Phytozome database (http://www.phytozome.net/) (access on 15 April 2018) and blasting the National Center for Biotechnology Information (http://www.ncbi.nlm.nih.gov/blast/) (access on 15 April 2018). All software and websites used are listed in Supplementary Table S2.

### 4.4. Plant Growth and Treatment

For the tissue-specific expression pattern analysis, different tissues (root, stem, leaf, flower and fruit) of *Malus hupehensis* (Pamp.) Rehd. *var. pinyiensis* were collected and used to quantify tissue-specific expression patterns of *MdCEPs* in apple. The apple trees were 12 years old and were from the Experimental Orchard of Shandong Institute of Fruit Tree Science (Tai’an, China).

For the induced expression pattern analysis, we selected wild varieties *Malus hupehesis* (Pamp.) Rehd. *var. pinyiensis* for various stress testing. Seeds were placed at 4 °C for 40 d, and the seedlings were transplanted into 50% Hoagland’s nutrient solution under greenhouse conditions (16/8 h light/dark cycles, 26 ± 1 °C). Uniformly developed seedlings at the three- or four-leaf stage were selected for stress treatments. For drought and salt treatments, seedlings were cultured in 50% Hoagland’s nutrient solution containing 20% PEG 6000 or 150 mM NaCl for 0, 3 or 24 h. For cold treatment, seedlings were cultured in 50% Hoagland’s nutrient solution at 4 °C. For IAA and ABA treatments, seedlings were spraying with 100 μM IAA or 100 μM ABA. Then, the roots and leaves were collected separately and rapidly frozen in liquid nitrogen.

### 4.5. RNA Extraction and qRT-PCR Analysis

Subsequently, the total RNA was extracted using Plant RNA Purification Reagent (Invitrogen, Carlsbad, CA, USA) as described in previous studies [21]. First-strand cDNA was synthesized using 2 μg of total RNA with PrimeScript First Strand cDNA Synthesis Kit (Takara, Dalian, China). qRT-PCR was performed using gene-specific primers and SYBR Premix Ex Taq (TaKaRa, Dalian, China) on a CFX96^TM^ Real-Time PCR Detection System (Bio-Rad, Hercules, California, USA) [31]. The *18s ribosomal RNA* was used as control. Three biological replicates were performed. The details of the designed primers are shown in Supplementary Table S3.

### 4.6. Gene Cloning

The ORF of *AtCEP1* was amplified from Arabidopsis Col-0 cDNA, and cloned into the pRI 101-AN DNA expression vector under the control of CaMV35S promoter, and ORFs of *MdCEP1*, *MdCEP^S^*, *MdCEP^G^* and *MdCEP^SG^* were amplified from *Malus hupehesis* (Pamp.) Rehd. *var. pinyiensis* cDNA using primers in Supplementary Table S3. Various plasmids were transformed into the *Agrobacterium tumefaciens* LBA4404 strain, respectively. The *Agrobacterium tumefaciens* was grown in LB medium supplemented with rifampicin (50 mg mL^−1^) and kanamycin (50 mg mL^−1^). For transformation of ‘Orin’ callus, 10-day-old callus were co-cultured with LBA4404 carrying various CEP vectors. The callus were co-cultured on MS medium containing 2, 4-D (1.5 mg L^−1^) and 6-BA (0.5 mg L^−1^) for 2 d at 24 °C. Subsequently, the callus were washed three times with sterile water and transferred to MS medium supplemented with carbenicillin (250 mg L^−1^) and kanamycin (50 mg mL^−1^) for transgene selection as described in previous research [32]. Transgenic callus and normal non-transgenic callus were transferred on MS medium for another 18 d under continuous dark conditions.

### 4.7. In vitro and in vivo Growth Inhibition Assays

Chemically synthesized peptides were purchased (≥ 95% purity, Sangon, Shanghai, China) (http://www.sangon.com/) (access on 28 April 2018). The sequences of relevant peptides were as follows: AtCEP1: HFRPTNPGNSPGVGH; MdCEP1: LGGIKDSGPSPGSGN; MdCEP^S^: LGGIKDSGPCPGSGN; MdCEP^G^: LGGIKDSGPSPGSAN; MdCEP^SG^: LGGIKDSGPCPGSAN. A dilution series of AtCEP1, MdCEP1, MdCEP^S^, MdCEP^G^ and MdCEP^SG^ in dimethyl sulfoxide (DMSO) were added into MS medium. Wild-type varieties *Malus hupehesis* (Pamp.) Rehd. *var. pinyiensis* were placed at 4 °C for 40 d, and then transplanted on MS medium with various CEP peptides under greenhouse conditions (16/8 h light/dark cycles, 26 ± 1 °C) for 2 weeks. Root length and lateral root number were examined and all the experiments were performed three times.

Another assay, 10-day-old ‘Orin’ callus with similar sizes were transferred on MS medium with various CEP peptides for another 18 d under continuous dark conditions. The fresh weight of callus was examined and all experiments were performed three times.

To further test whether the conserved serine was crucial for CEP function exertion, MdCEP1 and MdCEP^S^ subjected various seeds to treatment with higher land plants including *Nicotiana benthamiana*, *Raphanus sativus* L., Gramineae (*Setaria italica*) and *Setaria italica* (*Brassica campestris* L.). DMSO was used as the negative control. The sterile seeds were plated on MS medium with or without MdCEP1 or MdCEP^S^ and grown for 10 d.

To identify the function of *MdCEP1* in the N-demand signaling pathway in apple, 14-day-old apple seedlings were treated with 0.5 mM nitrates for 0, and 6 h, and the apple seedlings were subsequently collected and qRT-PCR assay was performed to detect the expression of *MdCEP1*. Moreover, the sterile apple seeds were plated on 1/2 MS medium with or without AtCEP1, MdCEP1 or MdCEP1^S^ and grown for 14 d. Then, the seedlings were collected and qRT-PCR assay was performed to detect the expression of NO_3^−^_ importer gene *MdNRT2.1*, the homologous of *AtNRT2.1*.

### 4.8. Data Availability 

All the data, method and materials that support the findings of this study are available from the corresponding author upon request.

## 5. Statistical Tests

All experiments were performed with at least three independent repetitions. One-way ANOVA Duncan’s test (*p* < 0.05) and independent sample *t*-test (* *p* < 0.05; ** *p* < 0.01; *** *p* <0.001) in Statistical Product and Service Solutions 24 (SPSS 24, IBM, Armonk, New York, USA) were used for statistical analysis.

## Supplementary Materials

Supplementary materials can be found at http://www.mdpi.com/1422-0067/20/3/775/s1. Supplementary Figure S1. Multiple sequence alignment of MdCEPs for apple. Figure S2. Signal peptide cleavage sites of MdCEPs predicated via SignalP 4.1 software. Supplementary Figure S3. The number of CEP genes in 39 plant species. Supplementary Figure S4. Multiple sequence alignment of 273 CEP members in 32 higher land plants, which a genome sequence was available. Supplementary Figure S5. Multiple sequence alignments of adjoining sequence between CEP domains in some higher land plants. Supplementary Table S1. Genome-wide analysis of MdCEPs in apple. Supplementary Table S2. References of bioinformatics software, website and databases. Supplementary Table S3. Primers used in this study.

## Abbreviations

CEP	C-terminally encoded peptide
PTMs	post-translationally modified peptides
CRPs	cysteine-rich peptides;
DMSO	dimethyl sulfoxide
qRT-PCR	quantitative real-time polymerase chain reaction
