# Supplementary material for: The Importance of Conserved Serine for C-Terminally Encoded Peptides Function Exertion in Apple
Source: Int J Mol Sci. 2019 Feb 12;20(3):775. doi: 10.3390/ijms20030775 (PMC6387203; doi:10.3390/ijms20030775)
Supplement: Supplementary file 1 [file ijms-20-00775-s001.pdf]

Supplementary Materials

Journal:
International Journal of Molecular Sciences

Title
The Importance of Conserved Serine for C-terminally Encoded Peptides Function Exertion in Apple

Authors:
Zipeng Yu[1,#], Yang Xu[1,2,#], Lin Liu[1], Yarong Guo[1], Xisen Yuan[1], Xinyu Man[1], Chang Liu[3], Guodong Yang[1], Jinguang Huang[1], Kang Yan[1], Chengchao Zheng[1], Changai Wu[1,Ψ] and Shizhong Zhang[1,Ψ]

[#] **Co-first author**
These authors contributed equally to this work.

[Ψ] **Corresponding author**
Shizhong Zhang and Changai Wu

Affiliation
[1] State Key Laboratory of Crop Biology, Shandong Agricultural University, Tai'an, Shandong 271018, China
[2] Shandong Peanut Research Institute, Shandong Academy of Agricultural Sciences, Qingdao 266100, China
[3] Entomology and Nematology Department, University of Florida, USA

[Ψ] **Corresponding author:**
Changai Wu: cawu@sdau.edu.cn
Shizhong Zhang: shizhong@sdau.edu.cn; Tel: +86-538-8241318

Supplementary Figure S1

```
MdCEP1   ...................................MALNKLTFIACSFLVSLLILTWEIQSINA.RYLKSRS.SELQNXP
MdCEP2   ......................................MGMRKLFNVLFIFLVLLLPMVATIRH.SNSKYQP........
MdCEP3   MKPIPHQHFFNLHKTHNPKSLNSPSLSPSLPPSLSTFLATMPRLTALALIFLVAMASSVSTLES.RKLLDVK........
MdCEP4   ........................................MTTKFFSFVLLLLLLNSTFIAVSA.RPLNIMKYRSSGFGA
MdCEP5   ........................................MTTKFFSFVLLLLLLNSTFIAVSA.RPLNIMKYRSSGFGA
MdCEP6   ........................................MPRLSAFALIFLVVMASFVSILES.RKLSDVKK.......
MdCEP7   .....................................MANGKLS...FLLLVLISSYGIISTEE.RFLKTDH........
MdCEP8   ..........................MEAKCSILLAFIACIEIVCIEGRPFLAKNKESINTIEA.NPKRELGEQVRQPAS
MdCEP9   ...................................MALNKLTLIACSFLVSLLISTWEIQSIDA.RPLKSRSKNELQNLP
MdCEP10  ........................................MTTKFFSFVLLLVLLNSTFIAVSA.RPLNIMKYKNSAVEA
MdCEP11  ..................................MATMAKAKLLCACVFLLALVLSSGIISTEA.RSMNTAD........
MdCEP12  ......................................MATSIFVITILMVALLLGQNFDLIAASRPLQIQP........

MdCEP1   IQNKPHENKTEDNAGHKSDLHGKSSTEAS.LVVSPP...PPQSGQVGGAAQPPPPPAHAVDDF.RPTAPGHSPGVGHSLQ
MdCEP2   .....PGATILKALRKRLIMEDFH........................AEADDYNGNGGDNDYYRRYGDVPSPGVGH...
MdCEP3   ..DDAENQKKVASLFLSALPKGKVPASTP..........SKKGHAVVVNEKLIARHLSVVQNR.ILAQSVPSPGVGH...
MdCEP4   TGGGFFDGLSLGAIKQSGPSPGEGHKLTN.....KQ...TLGGIKTSGPSPGSGNAFTNVEILGGIKDSGPSPGSGNKFT
MdCEP5   TGGGFFDGLSLGAIKQSGPSPGEGHKLTN.....KQ...TLGGIKTSGPSPXSGNAFTNVEILGGIKDSGPSPGSGNKFT
MdCEP6   ..DDAEYKKKEASLFLSALPKGNVPASTP..........SKKGHAVVINEKLIARHLSVEPNR.ILAQSVPSPGVGH...
MdCEP7   .....TNGGSTSMISHDNYLNSRRNVFEN...............ELS.DSVPPVPGYHSASDY.RPTTPGHSPGAGHSVG
MdCEP8   HHHIASPKTLHXTAHNFDDISGTHAAYKDDFRPTTPGNSPAVGHKFTKSGEYMGTKADDFVGAGRPTGPGHSPGVGHVX.
MdCEP9   IHTKTHKNKSEDNAGHKRDLHGKSTTEASSVVVSPP...PPQSDQVDGAAQLPPPPAHAVDDF.RPTAPGHSPGVGHSLQ
MdCEP10  ....FFDGLALGAIKESGPSPGEGHKFTN.....QQ...TLGGIKNSGPSLGTGDVFTNVETLGGIKNSGSSPITGNKFT
MdCEP11  .....PSNSNSGFIEPKNPSNSK..........................PSYTVVAVKEANAF.RPTTPGHSPGVGHGS.
MdCEP12  .....PVVIHKGSLSKPKPPSTAT......................FTIKRYKRTESSGGGDAF.RPTSPGHSPGVGNHQP
                                                                              sp   g

MdCEP1   N...........................................................................................
MdCEP2   ............................................................................................
MdCEP3   ............................................................................................
MdCEP4   NVETLGGIKDSGPTP.............GIKDSGPTPGTGNKFTNVETLGGIKEAGPSPGTGNKFTNVETLSGIKDSGPSPGTGNKFTDNTH
MdCEP5   NVETLGGIKDSGPTPXTGNKFTNVETLGGIKEAGPSPGTGNKFTNVETLGAIKDSGPSLGTGNKSTNVETLGGIKDSGPSPGTGNKFTDNIH
MdCEP6   ............................................................................................
MdCEP7   PQVEPNQ.....................................................................................
MdCEP8   XVQNAKPIP...................................................................................
MdCEP9   N...........................................................................................
MdCEP10  NVATLKGIKDSGPSP............................................GTGNKFTNVETLGGIKDSGPSPGQGNKFTDNIH
MdCEP11  PPVGC.......................................................................................
MdCEP12  PSTLL.......................................................................................
```

Supplementary Figure S1. Multiple sequence alignment of MdCEPs for apple. Overall conserved amino acids are in black. CEP domains are marked in open squares.

Supplementary Figure S2

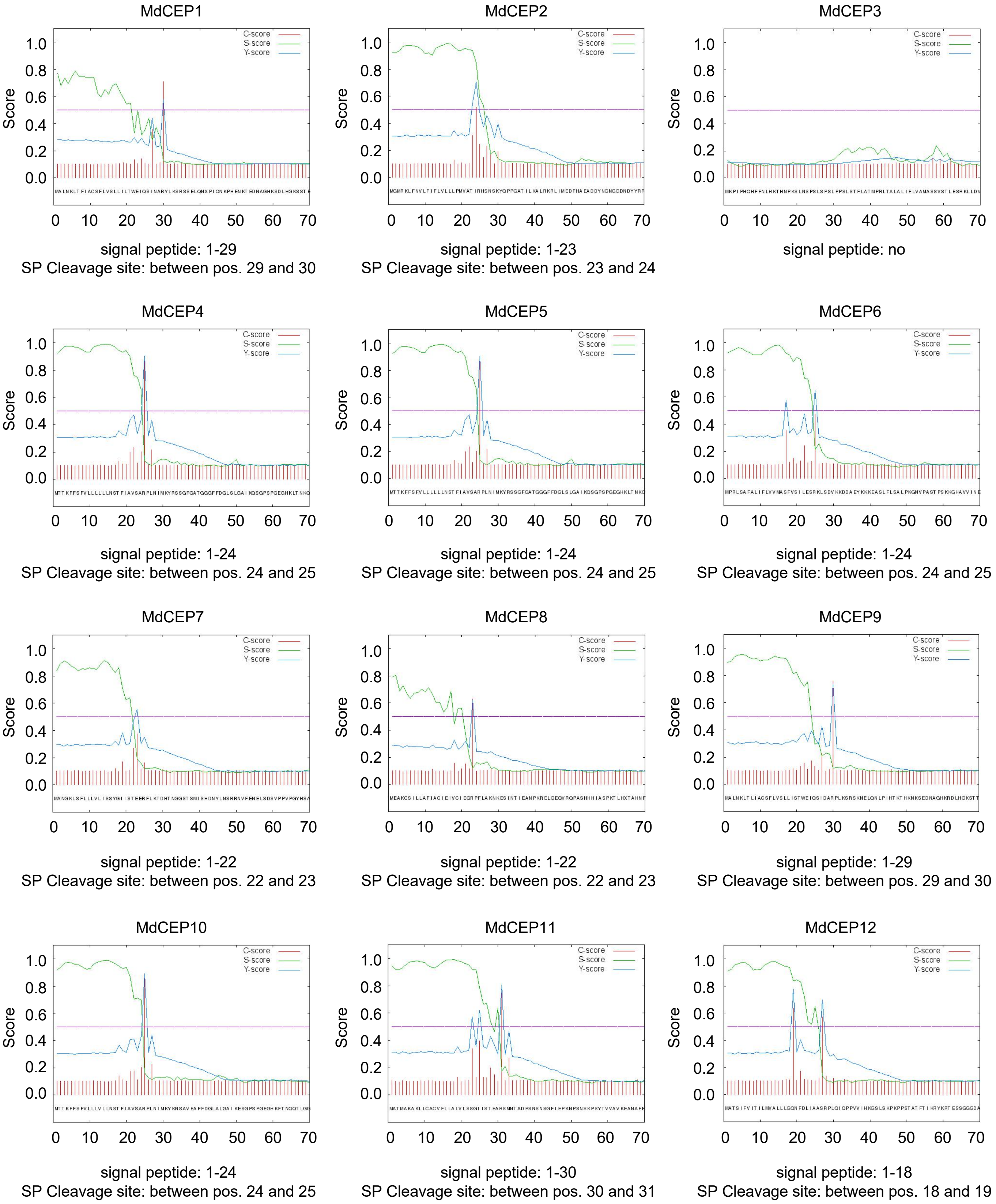

Supplementary Figure S2. Signal peptide cleavage sites of MdCEPs predicated via SignalP 4.1 software. The potential cleavage sites of various MdCEPs are shown in the bottom.

Supplementary Figure S3

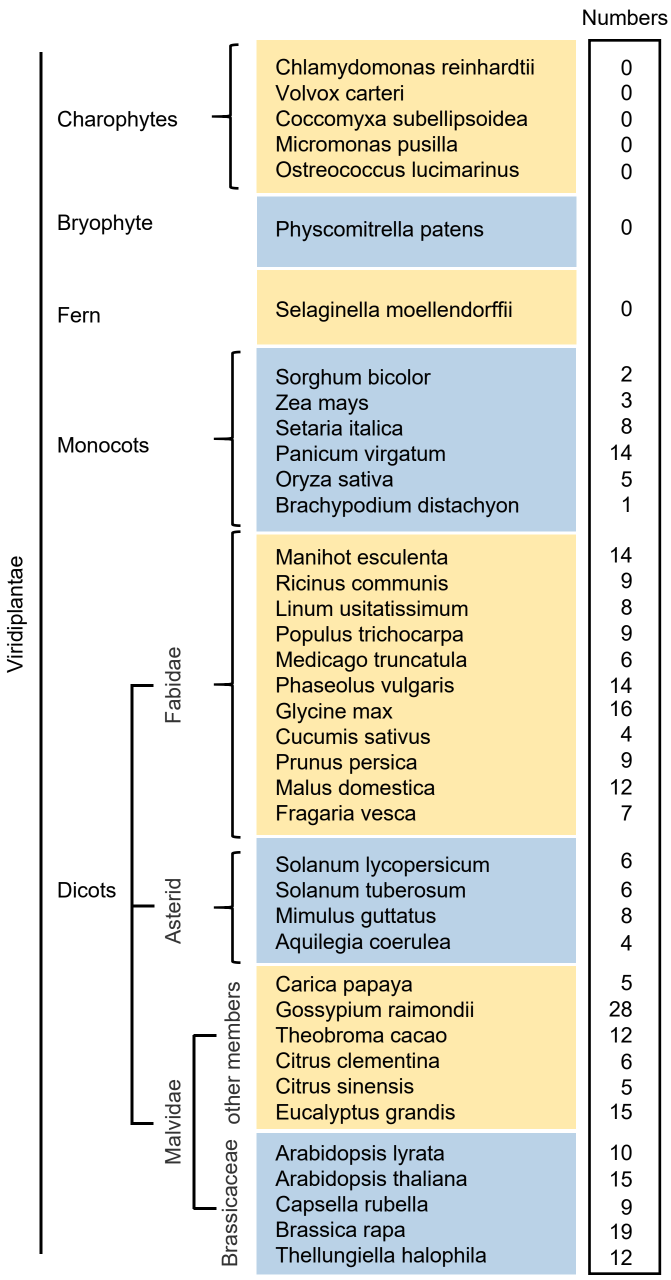

Supplementary Figure S3. The number of CEP genes in 39 plant species.
Phylogenetic tree indicating the presence or absence of CEP family members in the indicated species.

Supplementary Figure S4

Mes cassava4.1 017421m PACid 17967960	GSNVQA..PAPSPGASN	15
Mes cassava4.1 019941m PACid 17978185	IDRILQ..SVPSPGAGH	15
Mes cassava4.1 021376m PACid 17977760	.DRILINS.VPSPGAGH	15
Mes cassava4.1 022812m PACid 17987192	DYRVLQ..SVPSPGNGH	15
Mes cassava4.1 023188m PACid 17984324	.LGEIKES.GPSPGVGH	15
Mes cassava4.1 024116m PACid 17984762	.AF.RPTSPGHSPGVGH	15
Mes cassava4.1 025093m PACid 17960175	YYRRYG..DVPSPGIGH	15
Mes cassava4.1 027660m PACid 17971911	.DF.RPTVPGHSPGVGH	15
Mes cassava4.1 028634m PACid 17964410	.AF.RPTSPGHSPGAGH	15
Mes cassava4.1 028759m PACid 17964378	.AF.RPTSPGHSPGVGH	15
Mes cassava4.1 029507m PACid 17980573	.LGAIKQS.GPSPGVGH	15
Mes cassava4.1 030666m PACid 17983155	YYRRYG..DVPSPGVGH	15
Mes cassava4.1 031389m PACid 17992952	.DF.RPTEPGHSPGVGH	15
Mes cassava4.1 032405m PACid 17972083	IDRILR..SVPSPGVGH	15
Rco 27894.m000796 PACid 16799434	.GRVLAQS.VPSPGTGN	15
Rco 29676.m001611 PACid 16805358	.AF.LPPKTGSSPGIGH	15
Rco 29676.m001615 PACid 16805362	.DF.RPTAPGHSPGVGH	15
Rco 29729.m002349 PACid 16806998	YYRRYG..DVPSPGIGH	15
Rco 29801.m003091 PACid 16808941	.LEEVKKS.GPSPGIGH	15
Rco 29827.m002654 PACid 16810085	.AF.RPTAPGHSSGVGH	15
Rco 30026.m001466 PACid 16815851	IDRILQ..SVPSPGVGH	15
Rco 30026.m001467 PACid 16815852	..NFHKFYSVPSPGIGN	15
Rco 30170.m013702 PACid 16821549	.DF.RPTNPGHSPGAGH	15
Lus Lus10002969 PACid 23142128	.DF.RPTAPGHSPGVGH	15
Lus Lus10007309 PACid 23142478	NDRILR..SVPSPGMGH	15
Lus Lus10009345 PACid 23148889	.DF.RPTAPGHSPGVGH	15
Lus Lus10009347 PACid 23148880	.GF.RPTTPGHSPGVGH	15
Lus Lus10011512 PACid 23140441	.LGAIKES.GPSPGVGH	15
Lus Lus10019310 PACid 23141375	.LGAIKES.GPSPGVGH	15
Lus Lus10029256 PACid 23139795	IDRILR..SVPSPGMGH	15
Lus Lus10034745 PACid 23142246	.AY.RPTSPGHSPGVGH	15
Ptr POPTR 0001s30720.1 PACid 18236445	.ARAVKNS.GPSPGIGH	15
Ptr POPTR 0001s39620.1 PACid 18235960	TERFLL..AAPSPGIGN	15
Ptr POPTR 0004s05970.1 PACid 18226484	IDRILR..SVPSPGVGH	15
Ptr POPTR 0004s24290.1 PACid 18224779	.AF.RPTTPGHSPGVGH	15
Ptr POPTR 0005s18030.1 PACid 18207527	.DF.RPTTPGHSPGVGH	15
Ptr POPTR 0007s11230.1 PACid 18243387	.DF.RPTAPGHSPGVGH	15
Ptr POPTR 0007s11250.1 PACid 18243419	.AF.RPTNPGHSPGVGH	15
Ptr POPTR 0009s09850.1 PACid 18227516	.LQAVKKS.GPSPGIGH	15
Ptr POPTR 0011s06950.1 PACid 18232112	IDRILR..SVPSPGVGN	15
Mtr Medtr2g091770.1 PACid 17444308	.GRVLVSS.NPSPGAGH	15
Mtr Medtr4g135520.1 PACid 17459322	.DRVLIVS.VPSPGIGH	15
Mtr Medtr4g135670.1 PACid 17459338	.DRVLIVS.VPSPGIGH	15
Mtr Medtr5g017710.1 PACid 17462466	.AF.RPTTPGHSPGVGH	15
Mtr Medtr5g025790.1 PACid 17463336	.AF.RPTSPGHSPGVGH	15
Mtr Medtr8g086660.1 PACid 17481544	.AF.RPTSPGHSPGVGH	15
Pvu Phvulv091003416m PACid 23546681	.SF.RPTEPGHSPGVGH	15
Pvu Phvulv091010789m PACid 23548895	.DF.RPTDPGHSPGAGH	15
Pvu Phvulv091011675m PACid 23536742	.AF.RPTSPGHSPGVGH	15
Pvu Phvulv091012078m PACid 23534006	.AF.RPTTPGHSPGVGH	15
Pvu Phvulv091016399m PACid 23539039	.DF.RPTTPGHSPGVGH	15
Pvu Phvulv091016422m PACid 23539007	.AF.RPTTPGGSPGVGH	15
Pvu Phvulv091016515m PACid 23539113	.AF.RPTTPGNSPGVGH	15
Pvu Phvulv091016543m PACid 23539125	.AF.RPTTPGHSPGVGH	15
Pvu Phvulv091019763m PACid 23536020	.DF.QPTDPGHSPGAGH	15
Pvu Phvulv091022061m PACid 23543594	.ERLLVQS.VPSPGAGH	15
Pvu Phvulv091023136m PACid 23537216	.FRTLKSS.GPSPGIGH	15
Pvu Phvulv091025557m PACid 23550738	.DRVLLRS.VPSPGVGH	15
Pvu Phvulv091026499m PACid 23556901	FYRKHG..DIPSPGAGH	15
Pvu Phvulv091026517m PACid 23556935	.AF.RPTSPGHSPGVGH	15
Gma Glyma01g39160.1 PACid 16245686	.AF.RPTTPGHSPGVGH	15
Gma Glyma01g39170.1 PACid 16245687	.AF.RPTTPGNSPGVGH	15
Gma Glyma01g39180.1 PACid 16245688	.NF.RPTAPGHSPGVGH	15
Gma Glyma05g16420.1 PACid 16259024	.DF.KPTDPGHSPGVGH	15
Gma Glyma05g21360.1 PACid 16259188	.LGAMKDS.GPSPGVGH	15
Gma Glyma05g29340.1 PACid 16260047	.DRVLLRS.VPSPGVGH	15
Gma Glyma06g04110.1 PACid 16261649	.DF.QPTDPGHSPGAGH	15
Gma Glyma06g14900.1 PACid 16262990	.AF.RPTTPGHSPGVGH	15
Gma Glyma10g40880.1 PACid 16281683	.FRTLKSS.GPSPGVGH	15
Gma Glyma11g06090.1 PACid 16282854	.NF.RPTAPGHSPGVGH	15
Gma Glyma13g29870.1 PACid 16292048	.EQILLRS.VPSPGAGH	15
Gma Glyma14g08850.1 PACid 16294877	.DF.RPMDPGHSPGAGH	15
Gma Glyma16g16530.1 PACid 16302228	.AF.RPTCRGHSPGAGH	15
Gma Glyma17g20340.1 PACid 16306143	.AF.RPTTPGHSPGVGH	15
Gma Glyma17g20380.1 PACid 16306147	.AF.RPTTPGNSPGVGH	15
Gma Glyma17g36320.1 PACid 16307229	.DF.RPTDPGHSPGAGH	15
Csa Cucsa.057790.1 PACid 16954579	GDRVLV..SAPSPGIGH	15
Csa Cucsa.102340.1 PACid 16958856	.DF.RPTTPGHSPGVGH	15
Csa Cucsa.123520.1 PACid 16961609	.DF.RPTTPGHSPGAGH	15
Csa Cucsa.212840.1 PACid 16969396	YNRFLR..SVPSPGVGH	15
Ppe ppa013996m PACid 17660506	.DRILKEA.VPSPGVGH	15
Ppe ppa019651m PACid 17667080	.DF.RPTTPGHSPGVGH	15
Ppe ppa020187m PACid 17657463	.DF.RPTAPGHSPGVGH	15
Ppe ppa020556m PACid 17645603	.NY.RPTTPGHSPGAGH	15
Ppe ppa021649m PACid 17652075	.AF.RPTSPGHSPGVGH	15
Ppe ppa024356m PACid 17645973	.DF.RPTTPGNSPGVGH	15
Ppe ppa025356m PACid 17663365	.AF.RPTTPGHSPGVGH	15
Ppe ppa026968m PACid 17644867	.DRILAQA.VPSPGVGH	15
Fve mrna09052.1-v1.0-hybrid PACid 27265042	.DY.RPTEPGHSPGAGH	15
Fve mrna24020.1-v1.0-hybrid PACid 27255199	.LGSIKGS.GPSPGQGN	15
Fve mrna24064.1-v1.0-hybrid PACid 27257422	.DF.RPTAPGHSPGVGH	15
Fve mrna24066.1-v1.0-hybrid PACid 27260751	.AF.RPTTPGHSPGVGH	15
Fve mrna24067.1-v1.0-hybrid PACid 27254984	.DF.RPTTPGHSPGVGH	15
Fve mrna27636.1-v1.0-hybrid PACid 27268150	.AF.RPTSPGHSPGVGH	15
Fve mrna29711.1-v1.0-hybrid_PACid_27275107	NERFLQ..SVPSPGVGH	15
Aly 473139 PACid 16037455	VDRFLR..SVPSPGVGH	15
Aly 473891 PACid 16055390	.DF.RPTNPGNSPGVGH	15
Aly 483109 PACid 16062853	IYRRQG..DVPSPGIGH	15
Aly 889088 PACid 16063243	IYRRLE..SVPSPGVGH	15
Aly 900829 PACid 16037827	.TF.RPTAPGHSPGIGH	15
Aly 900830 PACid 16044513	.AF.RPTAPGHSPGVGH	15
Aly 902667 PACid 16061050	.AF.RPTHQGPSQGIGH	15
Aly 920038 PACid 16048311	.EF.RPTTPGNSPGIGH	15
Aly 920039 PACid 16054311	.AF.RPTNPGNSPGIGH	15
Aly 920041 PACid 16041467	.DF.RPTTPGHSPGIGH	15
Cru Carubv10010707m PACid 20893273	VERFLQ..SVPSPGVGH	15
Cru Carubv10012437m PACid 20892451	.DF.RPTSPGNSPGVGH	15
Cru Carubv10012478m PACid 20889145	IYRRLG..SVPSPGVGH	15
Cru Carubv10024377m PACid 20904277	IYRRQG..DVPSPAIGH	15
Cru Carubv10024395m PACid 20901584	.AF.RPTEPGHSPGVGH	15
Cru Carubv10025016m PACid 20902174	.NF.RPTVPGHSPGIGH	15
Cru Carubv10025501m PACid 20902024	.AF.RPTHQGPSQGIGH	15
Cru Carubv10027787m PACid 20911514	.SI.KPTTPGHSQGVGH	15
Cru Carubv10028621m PACid 20912421	.DF.RPTRPGNSPGIGH	15
Bra Bra000191 PACid 22711115	IYKRQG..DVPSPAIGH	15
Bra Bra004577 PACid 22686976	IYKRQG..DVPSPGIGH	15
Bra Bra005324 PACid 22686031	.AF.RPTHQGPSQGIGH	15
Bra Bra005442 PACid 22686799	VGRFLI..SAPSPGVGH	15
Bra Bra010838 PACid 22722136	VYRLLQ..SVPSPGVGH	15
Bra Bra016623 PACid 22724152	IHRQLG..SVPSPGVGH	15
Bra Bra016987 PACid 22719215	IYKRQG..DVPSPGIGH	15
Bra Bra017309 PACid 22720590	.AF.RPTHQGPSQGIGH	15
Bra Bra023015 PACid 22711471	.AF.RPTHQGPSQGIGH	15
Bra Bra026013 PACid 22697393	IYRQLG..SVPSPGVGN	15
Bra Bra030022 PACid 22703643	.DF.KPTTPGHSPGIGH	15
Bra Bra030133 PACid 22699493	VSRFLQ..SVPSPGVGH	15
Bra Bra031824 PACid 22688939	.DF.RPTTPGHSPGIGH	15
Bra Bra031827 PACid 22687899	.AF.RPTRPGNSPGIGH	15
Bra Bra032119 PACid 22720151	.AY.APTDPGNSPGIGH	15
Bra Bra032168 PACid 22720863	.TF.RPTVPGNSPGIGH	15
Bra Bra032301 PACid 22712773	VDRFLQ..SVPSPGVGH	15
Bra Bra038430 PACid 22723530	.AF.RPTGPGPSQGIGH	15
Bra Bra039200 PACid 22713613	.TF.RPTVPGHSPGIGH	15
Tha Thhalv10000591m PACid 20208839	.TF.RPTVPGHSPGIGH	15
Tha Thhalv10005231m PACid 20198406	.AF.RPTKPGNSPGIGH	15
Tha Thhalv10005479m PACid 20198659	.DF.GPTSPGNSPGIGH	15
Tha Thhalv10005488m PACid 20198906	.DF.RPTTPGHSPGIGH	15
Tha Thhalv10009335m PACid 20185879	IHRLLG..SVPSPGVGH	15
Tha Thhalv10009585m PACid 20185396	VDRLLQ..SVPSPGVGH	15
Tha Thhalv10009664m PACid 20187692	.AF.RPTLPGPSQGIGH	15
Tha Thhalv10009878m PACid 20186711	.AF.RPTVPGPSQGIGH	15
Tha Thhalv10011959m PACid 20184833	..FGRANDSGPSPGVGH	15
Tha Thhalv10012057m PACid 20184684	.DF.RPTNPGNSPGVGH	15
Tha Thhalv10017493m PACid 20180585	.AF.RPTHQGPSQGIGH	15
Tha Thhalv10017917m PACid_20180643	IYRRQG..DVPSPGIGH	15
Cpa evm.model.supercontig	.SY.RRQGDVPSPGVGH	15
Cpa evm.model.supercontig	.GF.RPTTPGISPGVGH	15
Cpa evm.model.supercontig_	.AF.RPTTPGHSPGVGH	15
Cpa evm.model.supercontig	.DF.RPTTPGHSPGVGH	15
Cpa evm.model.supercontig 7	IDPILQ..SVPSPGAGH	15

Gra Gorai.002G049200.1 PACid 26792439	.DRLLQSA.VPSPGAGH	15
Gra Gorai.002G049500.1 PACid 26794859	.DRLLQSA.IPSPGAGH	15
Gra Gorai.002G049600.1 PACid 26796693	IDRILQ..SVPSPGAGH	15
Gra Gorai.002G049700.1 PACid 26794205	.DRILQSS.HPSPGAGH	15
Gra Gorai.002G049800.1 PACid 26793324	.ERILQSS.HPSPGAGH	15
Gra Gorai.002G049900.1 PACid 26795843	.DRILQSS.SPSPGAGH	15
Gra Gorai.002G050000.1 PACid 26792726	.DRKLQTS.PPSPGNGH	15
Gra Gorai.002G050100.1 PACid 26796944	.DRILQKS.SPSPGNGH	15
Gra Gorai.003G021500.1 PACid 26797496	.DF.RPTTPGNSPGVGH	15
Gra Gorai.003G021800.1 PACid 26800507	.AF.RPTTPGHSPGVGH	15
Gra Gorai.003G021900.1 PACid 26798362	.DF.RPTTPGNSPGVGH	15
Gra Gorai.003G022000.1 PACid 26797807	.DF.RPTAPGHSPGVGH	15
Gra Gorai.003G022200.1 PACid 26797868	.DF.RPTAPGHSPGVGH	15
Gra Gorai.003G022300.1 PACid 26799828	.DF.RPTAPGHSPGVGH	15
Gra Gorai.003G022400.1 PACid 26798569	.DF.RPTGPGHSPGVGH	15
Gra Gorai.003G022600.1 PACid 26798810	.DF.RPTTPGNSPGVGH	15
Gra Gorai.004G176800.1 PACid 26774640	.AF.RPTTPGHSPGIGH	15
Gra Gorai.007G211200.1 PACid 26781242	.DF.RPTAPGHSPGAGH	15
Gra Gorai.008G074700.1 PACid 26814357	.DF.RRTSPGHSPGIGH	15
Gra Gorai.009G160500.1 PACid 26764647	IDRILQ..SVPSPGAGH	15
Gra Gorai.009G209700.1 PACid 26768310	.DL.HATTPGHSPGTGH	15
Gra Gorai.009G209800.1 PACid 26763590	.DV.YPTPRGHSPGAGH	15
Gra Gorai.009G275300.1 PACid 26770190	.AF.RSTTPGHSPGAGH	15
Gra Gorai.010G017400.1 PACid 26756912	.DF.RPTTPGHSPGAGH	15
Gra Gorai.010G161500.1 PACid 26758507	IDRILQ..SVPSPGAGH	15
Gra Gorai.011G106800.1 PACid 26811578	.LGGIKTS.GPSPGVGH	15
Gra Gorai.012G014300.1 PACid 26827614	FYRRQG..DVPSPGIGH	15
Gra Gorai.013G008000.1 PACid 26786574	FYRRQG..DVPSPGIGH	15
Tca Thecc1EG000584t1 PACid 27435472	.DF.RPTSPGHSPGVGH	15
Tca Thecc1EG000585t1 PACid 27435573	.DF.RPTTPGNSPGVGH	15
Tca Thecc1EG000586t1 PACid 27436771	.DF.RPTAPGHSPGVGH	15
Tca Thecc1EG016708t1 PACid 27448035	.AF.RPTSPGHSPGVGH	15
Tca Thecc1EG029645t1 PACid 27438142	IDRILQ..SVPSPGAGH	15
Tca Thecc1EG031987t1 PACid 27446390	IDRIMQ..SVPSPGGGH	15
Tca Thecc1EG031988t1 PACid 27444652	IDRILQ..SVPSPGAGH	15
Tca Thecc1EG031991t1 PACid 27445648	IDRILQ..SNPSPGSGH	15
Tca Thecc1EG031992t1 PACid 27444924	.DRVLEES.VPSPGGGN	15
Tca Thecc1EG031997t1 PACid 27444504	.IALSGE.SVPSPGAGN	15
Tca Thecc1EG033894t1 PACid 27459477	.DF.RPTTPGHSPGAGH	15
Tca Thecc1EG045547t1 PACid 27443274	FYRRQG..DVPSPGVGH	15
Csi orange1.1g038829m PACid 18122908	.AF.RPTSPGHSPGVGH	15
Csi orange1.1g039917m PACid 18125134	.LEAIKRS.GPSPGVGH	15
Csi orange1.1g040634m PACid 18124553	.DF.RPTAPGHSPGVGH	15
Csi orange1.1g045305m PACid 18125028	.AF.RPTTPGHSPGVGH	15
Csi orange1.1g048647m PACid 18110471	SDQILR..SVPSPGIGH	15
Ccl clementine0.9 026411m PACid 19270950	IDRILQ..SVPSPGIGH	15
Ccl clementine0.9 032010m PACid 19285040	.DF.RPTAPGHSPGVGH	15
Ccl clementine0.9 032865m PACid 19285197	.DF.RPTAPGHSPGVGH	15
Ccl clementine0.9 033186m PACid 19271760	.LKMRKMS.GPSPGIGH	15
Ccl clementine0.9 033497m PACid 19284793	.AF.RPTTPGHSPGVGH	15
Ccl clementine0.9 035096m PACid 19255717	.AF.RPTSPGHSPGVGH	15
Egr Eucgr.F00020.1 PACid 23580673	..KSSDGTGGFSPGMGH	15
Egr Eucgr.F04318.1 PACid 23585577	.DF.RPTEPGHSPGVGH	15
Egr Eucgr.F04319.1 PACid 23585578	.DF.RPTQPGHSPGVGH	15
Egr Eucgr.H01291.1 PACid 23590724	.ELIGEDS.GPSPGEGH	15
Egr Eucgr.H02229.1 PACid 23591542	.LGAIKQA.GPSPGEGH	15
Egr Eucgr.H02232.1 PACid 23591545	..RIKDSGPGPSPGEGH	15
Egr Eucgr.H02233.1 PACid 23591546	.LGAIKQA.GPSPSEGH	15
Egr Eucgr.H02234.1 PACid 23591547	.LGAIKQA.GPSPGEGH	15
Egr Eucgr.H02235.1 PACid 23591548	..RIKDSGPGPSPGEGH	15
Egr Eucgr.H02236.1 PACid 23591549	.LGAIKQA.GPSPGEGH	15
Egr Eucgr.H02237.1 PACid 23591550	.LGAIKQA.GPSPGEGH	15
Egr Eucgr.H02243.1 PACid 23591552	.LGAIKQA.RPSPGEGH	15
Egr Eucgr.I00948.1 PACid 23595456	.DF.RPTQPGHSPGVGH	15
Egr Eucgr.I00949.1 PACid 23595457	.DF.RPTEPGHSPGVGH	15
Egr Eucgr.I00950.1 PACid 23595458	.DF.RPTAPGHSPGVGH	15
Stu PGSC0003DMP400026186 PACid 24404023	.SLSGIKD.GPSPGVGH	15
Stu PGSC0003DMP400026192 PACid 24402207	..LGGIKD.GPSPGVGH	14
Stu PGSC0003DMP400026193 PACid 24403839	..LGGIKD.GPSPGVGH	14
Stu PGSC0003DMP400044189 PACid 24425840	.PS.PGHVDGHSPGIGH	15
Stu PGSC0003DMP400049579 PACid 24428490	IDRILR..SVPSPGVGH	15
Stu PGSC0003DMP400062761 PACid 24424565	.GF.GTSGPGHSPGIGH	15
Sly Solyc02g065040.1.1 PACid 27286301	.PS.PGHVDGHSPGIGH	15
Sly Solyc02g090590.1.1 PACid 27288319	.VD.AFSSPGHSPGIGH	15
Sly Solyc02g090610.1.1 PACid 27288096	.PP.PGHPDGHSPGIGH	15
Sly Solyc03g044180.1.1 PACid 27290005	..LGDIKD.GPSPGVGH	14
Sly Solyc03g044530.1.1 PACid 27289306	.DF.GPTGPGHSPGIGH	15
Sly Solyc03g044580.1.1 PACid 27289061	.GF.SPYGRGHSPGIGH	15
Mgu mgv11b016607m PACid 17688411	.DF.RPTTPGHSPGIGH	15
Mgu mgv1a015612m PACid 17696034	.AG.QPTTPGHSPGIGH	15
Mgu mgv1a016988m PACid 17679329	VDRILR..SVPSPGIGH	15
Mgu mgv1a017339m PACid 17696737	DGRSLG..SVPSPGNGH	15
Mgu mgv1a018810m PACid 17685127	.SY.RPTTPGHSPGIGH	15
Mgu mgv1a021644m PACid 17681094	VDRILT..SVPSPGVGH	15
Mgu mgv1a022124m PACid 17686602	.AF.RPTTPGNSPGMGH	15
Mgu mgv1a022966m PACid 17691044	.GFSYVKA.GPSPGEGH	15
Aco Aquca 015 00129.1 PACid 22042024	.DY.RPTAPGHSPGIGH	15
Aco Aquca 015 00130.1 PACid 22042091	.AF.RPTTPGHSPGIGH	15
Aco Aquca 039 00033.1 PACid 22062168	IDRILR..SVPSPGVGH	15
Aco Aquca 083 00041.1 PACid 22058359	.LAAVKNS.GPSPGEGH	15
Sbi Sb01g033370.1 PACid 1953117	.DV.RPSNPGHSPGIGH	15
Sbi Sb07g023150.1 PACid 1976350	.DS.RPTAPGNSPGIGN	15
Zma AC206165.3 FGT009 PACid 20820907	AARSMR..SVPSPGVGH	15
Zma GRMZM2G007969 T01 PACid 20871166	.DS.RPTAPGNSPGIGN	15
Zma GRMZM2G446946 T01 PACid 20834372	.DG.RPTAPGHSPGIGN	15
Sit Si014957m PACid 19693105	.DS.RPTAPGNSPGIGN	15
Sit Si023721m PACid 19698868	IEVADG..SVPSPGVGH	15
Sit Si024547m PACid 19700895	AARLMR..SVPSPGVGH	15
Sit Si031888m PACid 19713354	.DG.RPTAPGHSPGIGN	15
Sit Si032835m PACid 19711877	.DG.RPTAPGHSPGIGN	15
Sit Si038195m PACid 19679725	.DV.RPTTPTHSPGIGH	15
Sit Si038677m PACid 19682836	.DV.RPSTPRHSPGIGH	15
Sit Si038746m PACid 19684078	.DV.RPTTPTHSPGIGH	15
Pvi Pavirv00002044m PACid 23757424	TQVADG..SVPSPGVGH	15
Pvi Pavirv00009892m PACid 23825642	.DS.RPTAPGNSPGIGN	15
Pvi Pavirv00012112m PACid 23810237	.DT.RATDPGHSPGAGH	15
Pvi Pavirv00027227m PACid 23823988	AARVLG..SVPSPGVGH	15
Pvi Pavirv00027983m PACid 23815148	.DS.RPTAPGNSPGIGN	15
Pvi Pavirv00040661m PACid 23806253	.DV.RPTNPSHSPGIGH	15
Pvi Pavirv00042451m PACid 23814362	.DS.RPTAPGNSPGIGN	15
Pvi Pavirv00043494m PACid 23777395	.DG.RPTAPGHSPGIGN	15
Pvi Pavirv00043495m PACid 23777394	.DG.RPTAPGHSPGMGN	15
Pvi Pavirv00048689m PACid 23777482	.QT.DSTTPGHSPSIGH	15
Pvi Pavirv00054126m PACid 23804596	.DV.RPTNPSHSPGIGH	15
Pvi Pavirv00054493m PACid 23769669	AARVLG..SVPSPGVGH	15
Pvi Pavirv00056899m PACid 23826487	.DG.RATNPGHSPGIGN	15
Pvi Pavirv00060642m PACid 23757579	.DV.RPTNPSHSPGIGH	15
Osa LOC Os03g27690.1 PACid 21912942	.DV.RPTNPGHSPGIGH	15
Osa LOC Os05g11580.1 PACid 21941031	GWMPQG..SVPSPGIGH	15
Osa LOC Os05g11610.1 PACid 21941812	TAQLQG..SVPSPGIGN	15
Osa LOC Os08g37070.1 PACid 21891437	.DS.RPTAPGNSPGIGN	15
Osa LOC Os09g28780.1 PACid 21925812	.DG.RPTSPGHSPGIGN	15
Bdi Bradi1g60570.1_PACid_21816944	.GV.RPSNPGHSPGIGH	15
Consistant	S	

Supplementary Figure S4.
Multiple sequence alignment of 273 CEP members in 32 higher land plants, which a genome sequence was available. And Ser[10] was evolutionarily conserved in these 32 species.

Supplementary Figure S5

Species	Linker between CEP domains	WebLoGo	Sequence
Malus domestica	>KLTNKQT >KFTNVET<br>>AFTNVET >KFTNVET<br>>KFTNVET >KSTNVET<br>>KFTNVET >KFTDNIH<br>>KFTNVET >KFTNQQT<br>>KFTDNTH >VFTNVET<br>>KLTNKQT >KFTNVAT<br>>AFTNVEI >KFTNVET<br>>KFTNVET >KFTDNIH	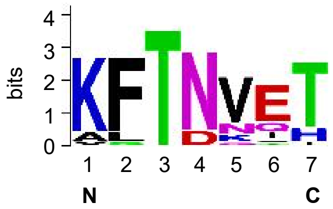	KFTNVET
Eucalyptus grandis	>KFTNSET >KFTNSKT<br>>KFTNSET >KFTNSET<br>>KFTNSET >KFTNSET<br>>KFTNSKT >KFTNSET<br>>KFTNSET >KFTNSET<br>>KFTNSET >KFTNLET<br>>KFTNSET >KFTNSRT<br>>KFTNSET >KFTNSRT<br>>KFTNLET >KFTNSET	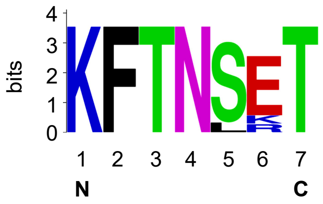	KFTNSET
Fragaria vesca	>KFTDQRT<br>>KVTNVET<br>>SFTNVDT<br>>KFTNVGT	The number of sequences is too small to establish the logo	KFTNV_T
Solanum tuberosum	>KFTNSQT<br>>KFTNSQT<br>>KFTNSQI	The number of sequences is too small to establish the logo	KFTNSQT
Ricinus communis	>KYKNLQT	The number of sequences is too small to establish the logo	KYKNLQT
Citrus sinensis	>KYNDRNT<br>>KLTDNHT	The number of sequences is too small to establish the logo	K__D__T
Citrus clementina	>KYENFKT	The number of sequences is too small to establish the logo	KYENFKT
Solanum lycopersicum	>KFTNNQT	The number of sequences is too small to establish the logo	KFTNNQT
Mimulus guttatus	>KFTNVET<br>>KLTDTKT	The number of sequences is too small to establish the logo	K_T___T
Populus trichocarpa	>KYKNFQS<br>>KYENFQT	The number of sequences is too small to establish the logo	KY_NFQ_
Glycine max	>KFTNSET<br>>QFTNSET<br>>KFTNSET<br>>RLKNLQN	The number of sequences is too small to establish the logo	KFTNSET
All of above plants	All of above sequences	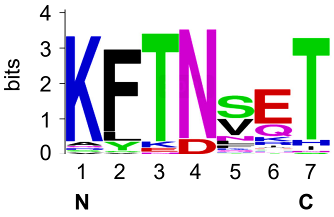	KFTNSET

Supplementary Figure S5. Multiple sequence alignments of adjoining sequence between CEP domains in group 2. The adjoining sequences (KFTNVET) between CEP domains in group 2 showed high amino acid similarity (KFTNVET), indicating that the proteolytic processing might be identical in some higher land plants.

Supplementary Table S1. Genome-wide analysis of MdCEPs in apple.

Gene id	protein length	pI	Mw	CDS length	exon no.	Genomic location
MDP0000143462	191	9.52	19277.57	576	2	MDC035751.16:9289..9911
MDP0000143463	204	9.49	20643.54	615	1	MDC035751.16:12043..12657
MDP0000146517	96	5.87	10384.43	291	1	MDC001634.432:141..431
MDP0000187706	81	9.1	9183.6	246	1	MDC010707.221:2870..3115
MDP0000213875	119	7.01	12673.43	360	1	MDC017493.407:10804..11163
MDP0000254055	96	9.92	10378.21	291	1	MDC029559.30:7325..7615
MDP0000306151	156	9.44	16109.15	471	1	MDC018097.432:5007..5477
MDP0000375783	89	8.99	9088.43	270	1	MDC018728.84:5624..5893
MDP0000770985	92	10.62	9746.36	279	1	MDC005909.470:7191..7469
MDP0000804928	135	10.46	14510.03	408	1	MDC015524.135:32478..32885
MDP0000886456	141	7.82	15122.71	426	1	MDC022708.143:7353..7778
MDP0000886459	121	7.98	12978.62	366	1	MDC022708.143:15790..16155

Supplementary Table S1. The detailed information including protein length, molecular weight, isoelectric point (pI), CDS length, number of exons and genomic location are shown in the table.

Supplementary Table S2. References of bioinformatics software, website and databases.

**Bioinformatic software and databases**	**Websites**	**References**
Phytozome	http://www.phytozome.net/	Goodstein et al. 2012
ExPASy Proteomics Server	http://expasy.org/	Gasteiger et al. 2003
Plant-CARE Server	http://bioinformatics.psb.ugent.be/webtools/plantcare/html/	Jia et al. 2015
MUSCLE program	http://www.drive5.com/muscle/	Edgar 2004
MEGA7	http://www.megasoftware.net/	Tamura. et al. 2011
Gene Structure Display Server	http://gsds.cbi.pku.edu.cn/	Hu. et al. 2015
Treeview	http://gijgo.com/tree/	Zhang. et al. 2013
I-TASSER	http://zhanglab.ccmb.med.umich.edu/I-TASSER/	Yang. et al. 2015
BAR	http://bar.utoronto.ca/	Kiana Toufighi. et al. 2005
DNAMan	https://www.lynnon.com/pc/framepc.html	Jyrki Kaukonen. et al. 2000
TAIR10	https://www.arabidopsis.org/	Tanya Z. et al. 2015
Genevestigator	https://genevestigator.com/gv/	Hruz T. et al. 2008
TMHMM Server v. 2.0	http://www.cbs.dtu.dk/services/TMHMM/	S. Moller. et al. 2001
SUBA4	http://suba.live/	Hooper CM. et al. 2017
SignalP4.1	http://www.cbs.dtu.dk/services/SignalP/	Nielsen H. 2017
PLAZA comparative genomics tool	http://bioinformatics.psb.ugent.be/plaza/	Van Bel. et al. 2017
Uniprot databases	http://uniprot.org/blast/	Wu J. et al. 2014
Camelina sativa database	http://www.camelinadb.ca/	Kagale, S. et al. 2014
Brassica Database	http://brassicadb.org/brad/index.php/	Cheng F. et al. 2011
GDDH13 Version 1.1	https://iris.angers.inra.fr/gddh13/the-apple-genome downloads.html	Nicolas Daccord. et al. 2017

Supplementary Table S3. Primers used in this study.

Purpose	Name	Accession number of genes	Sequence (5′-3′)
Transgenic callus<br><br>Vector,<br>pRI 101-AN DNA	AtCEP1 F	AT1G47485	GTCGACATGGGAATGTCGAATAGGTCAGT
	AtCEP1 R	AT1G47485	GGATCCTCAATGTCGCCCGTTAGAGT
	MdCEP1 F	MDP0000213875	GTCGACATGGCGACCATGGCCAAA
	MdCEP1 F	MDP0000213875	GGATCCTCAACATCCAACAGGAGGG
	MdCEP^{S} F	MDP0000213875	GTCGACATGGCGACCATGGCCAAA
	MdCEP^{S} F	MDP0000213875	GGATCCTCAACATCCAACAGGAGGGCTTCCATGACCAACACCAG GACAGTGGCCTGGT
	MdCEP^{G} F	MDP0000213875	GTCGACATGGCGACCATGGCCAAA
	MdCEP^{G} F	MDP0000213875	GGATCCTCAACATCCAACAGGAGGGCTTCCATGTGCAACACCAGG ACTGTGG
	MdCEP^{SG} F	MDP0000213875	GTCGACATGGCGACCATGGCCAAA
	MdCEP1^{SG} F	MDP0000213875	GGATCCTCAACATCCAACAGGAGGGCTTCCATGTGCAACACCAGG ACAGTGGCCTGGT
qRT-PCR<br>in<br>*Malus domestica*	Md CEP1 F	MDP0000213875	ACTGAAGCATCATTAGTTGTATC
	Md CEP1 R	MDP0000213875	GCCTGAAGTCATCTACGG
	Md CEP2 F	MDP0000187706	ATCAGGCACAGTAATAGCA
	Md CEP2 R	MDP0000187706	TAATAGTCGTTATCTCCACCAT
	Md CEP3 F	MDP0000804928	AAACCCACAATCCCAAATC
	Md CEP3 R	MDP0000804928	TAGAGTTGACACAGATGAGG
	Md CEP4 F	MDP0000143462	TCGGAGGGATTAAGGATTC
	Md CEP4 R	MDP0000143462	CCACCAAGAGTCTCAACA
	Md CEP5 F	MDP0000143463	AGCGATTAAGGACTCTGG
	Md CEP5 R	MDP0000143463	TTATTGATGGATGTTGTCTGTAA
	Md CEP6 F	MDP0000254055	AGGCTTCTCTGTTTCTAAGT
	Md CEP6 R	MDP0000254055	GATTTGGTTCAACGCTAAGA
	Md CEP7 F	MDP0000146517	ATCAGCCATGACAATTATCTC
	Md CEP7 R	MDP0000146517	CTACTTGAGGTCCTACAGAA
	Md CEP8 F	MDP0000886456	TGGCAAAGAACAAAGAGTC
	Md CEP8 R	MDP0000886456	GACTAGCAATGTGGTGATG
	Md CEP9 F	MDP0000886459	TCATCTCAACCTGGGAAAT
	Md CEP9 R	MDP0000886459	GCATTATCCTCCGATTTGTT
	Md CEP10 F	MDP0000306151	TGGAGCCATCAAGGAATC
	Md CEP10 R	MDP0000306151	CCACCTAGAGTCTCAACATT
	Md CEP11 F	MDP0000375783	GTTCTCTCTTCTGGAATCATTT
	Md CEP11 R	MDP0000375783	CTGGGTTTGGAATTAGATGG
	Md CEP12 F	MDP0000770985	AGCGTTACAAGAGGACTG
	Md CEP12 R	MDP0000770985	GTACTAGGTGGCTGATGATT
	18s rRNA F	DQ912698.1 (gene bank)	ACACGGGGAGGTAGTGACAA
	18s rRNA R	DQ912698.1 (gene bank)	CCTCCAATGGATCCTCGTTA
	MdNRT2.1 F	MD11G1141700 F	GTGTGCTGTACTCTTCCT
	MdNRT2.1 R	MD11G1141700 R	CACGCTCAGACCTACTATT
